# Metabolic, microbial, and pharmacological stimuli elicit distinct lipidomic and cytokine responses in the human placenta

**DOI:** 10.1093/molehr/gaag016

**Published:** 2026-03-07

**Authors:** Fiona Kumnova, Oleksandr Kozlov, Eva Cifkova, Eva Trckova, Alba Gonzalez, Michaela Medkova, Nida Cavdarbasha, Cilia Abad, Miroslav Lisa, Lukas Cerveny, Frantisek Staud, Rona Karahoda

**Affiliations:** Department of Pharmacology and Toxicology, Faculty of Pharmacy in Hradec Kralove, Charles University, Hradec Kralove, Czech Republic; Department of Chemistry, Faculty of Science, University of Hradec Kralove, Hradec Kralove, Czech Republic; Department of Chemistry, Faculty of Science, University of Hradec Kralove, Hradec Kralove, Czech Republic; 3rd Department of Internal Medicine – Metabolic Care and Gerontology, University Hospital Hradec Kralove, Hradec Kralove, Czech Republic; Department of Pharmacology and Toxicology, Faculty of Pharmacy in Hradec Kralove, Charles University, Hradec Kralove, Czech Republic; Department of Pharmacology and Toxicology, Faculty of Pharmacy in Hradec Kralove, Charles University, Hradec Kralove, Czech Republic; Department of Pharmacology and Toxicology, Faculty of Pharmacy in Hradec Kralove, Charles University, Hradec Kralove, Czech Republic; Department of Pharmacology and Toxicology, Faculty of Pharmacy in Hradec Kralove, Charles University, Hradec Kralove, Czech Republic; Department of Chemistry, Faculty of Science, University of Hradec Kralove, Hradec Kralove, Czech Republic; Department of Pharmacology and Toxicology, Faculty of Pharmacy in Hradec Kralove, Charles University, Hradec Kralove, Czech Republic; Department of Pharmacology and Toxicology, Faculty of Pharmacy in Hradec Kralove, Charles University, Hradec Kralove, Czech Republic; Department of Pharmacology and Toxicology, Faculty of Pharmacy in Hradec Kralove, Charles University, Hradec Kralove, Czech Republic

**Keywords:** placenta, lipidomics, inflammation, cytokines, glucose, ceramides, triglycerides, metformin

## Abstract

The placenta integrates metabolic and immune functions essential for fetal development, and disruptions in lipid metabolism and inflammatory signaling have been implicated in pregnancy complications such as gestational diabetes mellitus (GDM), infection-associated inflammation, and preterm birth. To examine how distinct clinically relevant stressors shape these processes, we investigated placental explant responses to three representative exposures. These included high glucose (metabolic stress relevant to diabetes/GDM), lipopolysaccharide (LPS; microbial-inflammatory stress relevant to infection/chorioamnionitis), and metformin (pharmacological exposure in treated pregnancies). Human term placental explants were cultured under controlled *ex vivo* conditions and exposed to these stimuli. Lipidomic profiling was performed using supercritical fluid chromatography–mass spectrometry (SFC–MS), and inflammatory responses were assessed at gene, protein, and cytokine levels by qRT-PCR, western blotting, and ELISA. Explants preserved the lipid complexity of intact placenta and secreted defined lipid species, including free fatty acids, phospholipids, and sterols, indicating selective export. High glucose induced only modest cytokine responses, suggesting that acute exposure alone is insufficient for robust inflammasome activation. In contrast, LPS triggered pronounced lipid remodeling with consistent ceramide accumulation, alongside strong upregulation of interleukin 1 beta (IL1B), interleukin 6 (IL6), and tumor necrosis factor alpha (TNF) transcripts and protein release, supporting a role for ceramides in innate immune activation. Metformin displayed modest downregulation of inflammatory transcripts and lipid remodeling under basal conditions but paradoxical potentiation of LPS-induced cytokine release. These findings show that human placental explants retain key metabolic and immune functions and reveal distinct stimulus-specific signatures, offering insight into placental adaptation to metabolic, microbial, and pharmacological stressors relevant to pregnancy complications.

## Introduction

The placenta is a metabolically dynamic and immunologically active organ that serves as the critical interface between mother and fetus throughout pregnancy. It performs essential functions in nutrient and gas exchange, hormone production, and immune tolerance (Staud and Karahoda, 2018). Among these, the regulation of lipid metabolism is fundamental to both placental and fetal development (Thornburg *et al.*, 2019; Bidne *et al.*, 2022). Lipids not only form structural components of cellular membranes but also act as signaling molecules, mediators of inflammation, and energy reservoirs (Zhang *et al.*, 2018). Alterations in placental lipid homeostasis have been implicated in a wide range of pregnancy complications, including gestational diabetes mellitus (GDM), preterm birth, preeclampsia, and fetal growth restriction (Delmis, 1989; Brown *et al.*, 2016; Khaire *et al.*, 2021; Steinhauser *et al.*, 2021; Jiang *et al.*, 2022). Understanding how the placental lipidome responds to physiological and pathological stimuli is thus of central importance to maternal–fetal health.

Increasing evidence indicates that maternal metabolic disturbances, infections, and pharmacological interventions each have the capacity to perturb lipid homeostasis (Lu *et al.*, 2016; Huhtala *et al.*, 2020; Zhan *et al.*, 2021; Frankevich *et al.*, 2023). These influences are relevant across multiple complicated pregnancies, including metabolic disorders such as GDM and inflammatory or infection-driven conditions, which may occur independently or in combination (Romero *et al.*, 2014; Pantham *et al.*, 2015). Importantly, lipid metabolism and inflammation are tightly linked processes: lipid species can function as signaling molecules that modulate inflammation, while inflammatory stimuli in turn can alter lipid biosynthesis, storage, and degradation (Glass and Olefsky, 2012; Arifin and Falasca, 2016; Zhang *et al.*, 2018; Anand, 2020). This bidirectional relationship adds complexity to our understanding of placental function under stress conditions and highlights the need to study both processes in parallel.

Chronic hyperglycemia, as occurs in metabolic disorders, including GDM, has been associated with shifts in placental lipid transport and storage, potentially leading to lipotoxicity and contributing to adverse pregnancy outcomes (Ryckman *et al.*, 2015). Elevated glucose concentrations have also been shown to alter trophoblast function and cytokine production, reflecting the proinflammatory potential of metabolic stress (Han *et al.*, 2015; Corrêa-Silva *et al.*, 2018; Heim *et al.*, 2018; Tao *et al.*, 2024). Beyond cytokine induction, hyperglycemia has been shown to activate the NOD-, LRR-, and pyrin domain-containing protein 3 (NLRP3) inflammasome (Han *et al.*, 2015; Corrêa-Silva *et al.*, 2018; Jimenez-Escutia *et al.*, 2023), a central mediator of sterile inflammation that is expressed and functionally active in the placenta (Megli *et al.*, 2021). These findings suggest that metabolic stressors may alter placental function through combined effects on lipid and immune pathways.

Microbial signals also represent important triggers of placental inflammation, particularly in the context of intrauterine infection and chorioamnionitis, which are strongly associated with adverse outcomes, including preterm birth (Romero *et al.*, 2014). Lipopolysaccharide (LPS), a component of Gram-negative bacterial cell walls, activates Toll-like receptor 4 (TLR4) and is widely used as a reproducible experimental model of infection-driven placental inflammation (Abad *et al.*, 2024). In addition to inducing cytokine production (Abad *et al.*, 2024), LPS has been reported to alter lipid metabolic pathways (Lien *et al.*, 2020), but the extent to which it drives coordinated changes in both lipid profiles and cytokine dynamics in human placental tissue remains incompletely defined. Clarifying these responses is essential for understanding how microbial-inflammatory stimuli reshape placental lipid–immune crosstalk.

Metformin is a widely used antidiabetic agent that is increasingly prescribed during pregnancy for the management of type 2 diabetes and GDM (Lindsay and Loeken, 2017; Campbell, 2020; McEvoy *et al.*, 2025). It is traditionally known for its anti-hyperglycemic effects through enhanced insulin sensitivity and reduced hepatic gluconeogenesis. However, emerging evidence suggests that metformin also impacts lipid metabolism by promoting fatty acid oxidation and reducing lipid accumulation (Hur and Lee, 2015; Owen *et al.*, 2021). In addition, metformin has been shown to influence immune signaling in a context-dependent manner, with both anti-inflammatory and pro-inflammatory effects reported across different tissues and models (Xian *et al.*, 2021; Ryssdal *et al.*, 2023; Hu *et al.*, 2024; Huang *et al.*, 2024). Given its increasing clinical use during pregnancy, understanding the direct effects of metformin on placental lipid and immune responses is of considerable translational interest.

Taken together, high glucose, LPS, and metformin represent clinically relevant metabolic, microbial, and pharmacological stimuli that converge on pathways central to placental function. Studying their effects provides an opportunity to dissect how the placenta integrates diverse external signals across lipid metabolism and immune regulation. Each stimulus models a distinct stress context relevant to pregnancy complications, including GDM, infection-associated inflammation (e.g. chorioamnionitis), and preterm birth. To this end, we employed human placental explants, which preserve the cellular heterogeneity and architecture of the native tissue while enabling controlled experimental manipulation (Miller *et al.*, 2005). We aimed to characterize lipid composition and secretion under culture conditions and to determine how these stimuli differentially modulate placental lipidomic profiles and inflammatory responses, including cytokine expression and release.

## Materials and methods

### Chemicals and reagents

d-(+)-Glucose, d-Mannitol, LPS from *Escherichia coli* O111: B4, and thiazolyl blue tetrazolium bromide (MTT) were obtained from Sigma-Aldrich, St. Louis, MO, USA. Bicinchoninic acid (BCA) assay reagents were obtained from Thermo Fisher Scientific, Waltham, MA, USA. Tri Reagent solution was acquired from the Molecular Research Centre, Cincinnati, OH, USA. LC–MS grade methanol, propan-2-ol, and HPLC grade methyl tert-butyl ether, chloroform, and n-hexane were purchased from Honeywell, Charlotte, NC, USA. Ammonium formate and water (all LC–MS grade) were obtained from Merck, Darmstadt, Germany. Carbon dioxide (4.5 grade, 99.995%) was purchased from Messer Group GmbH, Bad Soden, Germany. Lipid class internal standards such as fatty acid 14:0, diacylglycerol 12:1/0:0/12:1, triacylglycerol 19:1/19:1/19:1 (Nu-ChekPrep, Elysian, MN, USA), cholesteryl ester 16:0[D7], cholesterol[D7], ceramide d18:1/12:1, phosphatidylcholine 14:0/14:0, phosphatidylethanolamine 14:0/14:0, sphingomyelin d18:1/12:0 (Avanti Polar Lipids, Alabaster, AL, USA) were used for the quantification of lipid species. All other chemicals used were of analytical grade.

### Human term placental explant isolation and culture

Human term placentas (n = 28) were obtained from uncomplicated singleton pregnancies delivered by caesarean section between 38 and 40 weeks of gestation. Women with preeclampsia, diabetes mellitus, GDM, gestational hypertension, or pregnancies complicated by fetal structural malformations, chromosomal abnormalities, fetal growth restriction, vaginal bleeding, and/or signs of fetal hypoxia were excluded from the study. Demographic characteristics of the study participants are summarized in Supplementary Table S1. Placentas were collected immediately after delivery at the University Hospital in Hradec Kralove, Czech Republic. All experiments were conducted in compliance with the Declaration of Helsinki, with written informed consent obtained from all participants. The study was approved by the University Hospital Research Ethics Committee (reference number: 202205 P09).

Cotyledon fragments were carefully separated by dissection from the placenta, followed by the removal of the chorionic plate and decidua (Abad *et al.*, 2024). The villous tissue was further cut into smaller pieces, each about 30 mg. Randomly selected villous tissue was cleaned of large blood vessels and clots, rinsed with cold sterile saline, and placed into 12-well plates. Each well contained 2 ml of DMEM Low glucose (5.5 mM) with l-Glutamine and Sodium Pyruvate (Capricorn Scientific, Ebsdorfergrund, Germany), supplemented with 10% fetal bovine serum (FBS), 100 U/ml penicillin, 0.1 mg/ml streptomycin, and 2.5 µg/ml amphotericin B. Three explants, totaling around 100 mg of tissue, were placed in each well. The explants were incubated in an environment with 20% O_2_ (for lipidomic analysis) or 8% O_2_ (for all other analyses), 5% CO_2_, and 87% N_2_ at 37°C in a sterile incubator. After 4 h of initial incubation, the medium was replaced, and the explants were allowed to stabilize and recover from the isolation process for 18–24 h before initiating experiments.

Explants incubated in standard culture medium containing 5.5 mM glucose served as baseline controls. Osmotic controls were included using mannitol at concentrations equivalent to glucose treatments. For lipidomic analyses, explants were incubated for 24 h in the absence or presence of high glucose (25 mM), metformin (100 µM), or LPS (10 µg/ml). To minimize exogenous lipid interference, delipidated FBS (Capricorn Scientific) was used in place of standard FBS in these experiments. For inflammatory response studies, explants were cultured for various durations (1 to 72 h) with or without high glucose (35 mM), metformin (1 mM), or LPS (10 µg/ml). To assess concentration-dependent effects, separate experiments were conducted using increasing concentrations of glucose (5, 10, 15, 25, and 35 mM) and LPS (0.1, 1, and 10 µg/ml), with sample collection at the 12-hour time point. Finally, to assess the modulatory effects of metformin on LPS-induced inflammation, placental explants were pretreated with metformin (0.1–1000 µM) for 24 h and then co-exposed to metformin and LPS (1 µg/ml) for an additional 6 h. Samples were collected post-LPS treatment and compared to control explants treated with LPS alone (1 µg/ml, 6 h). In parallel, to evaluate the time-dependent effects of metformin alone, explants were treated with 1 mM metformin, and samples were collected at 1, 3, 6, 12, 48, and 72 h.

For all experiments extending beyond 24 h, the culture medium was replenished every 24 h. At the end of each experiment, both explant tissue and culture supernatants were collected. Supernatants were centrifuged at 10 000*g* for 10 min to remove debris, and all samples were stored at −80°C until further analysis.

### Viability and integrity evaluation of placental explants

Explant viability was assessed using the MTT assay as previously described (Abad *et al.*, 2024). Explants were rinsed with warm Opti-MEM (Gibco, Thermo Fisher Scientific, Waltham, MA, USA) and incubated with 0.5 mg/ml MTT solution (Sigma-Aldrich) at 37°C for 45 min in the dark. Formazan crystals formed were dissolved by transferring the explants to wells containing 1 ml DMSO and shaken at room temperature for 5 min. The formazan content, indicative of cell viability, was quantified by measuring absorbance (Abs) at 570 nm and 690 nm, with results calculated as the difference (Abs 570—Abs 690) and normalized to tissue weight (mg). On the other hand, explant integrity was evaluated by measuring lactate dehydrogenase (LDH) release into the culture medium, as previously described (Abad *et al.*, 2024). LDH activity was determined using a colorimetric assay kit (Sigma-Aldrich) following the manufacturer’s instructions. The LDH activity in the culture medium was normalized to the explant weight (mg). Positive controls included explants treated with lysis buffer (20 mM Tris-HCl, 150 mM NaCl, 12.7 mM EDTA, 1 mM EGTA, 4 mM Na_4_P_2_O_7_, 1 mM Na_3_VO_4_, 1% Triton X-100, protease inhibitors, pH 6.8) for 15 min at 37°C to maximize LDH release. These tests confirmed that the highest tested concentrations and incubation times of glucose, mannitol, and LPS did not compromise explant viability or integrity (Supplementary Fig. S1).

### Sample preparation for SFC–MS lipidomic analysis

Total lipid extracts of placental explants and cultivation media were prepared using a methyl *tert*-butyl ether–methanol–water system. Briefly, 20 mg of explant tissue was homogenized with 350 µl of methanol using a FastPrep-24 5G instrument (MP Biomedicals, Irvine, CA, USA) and zirconium oxide beads (type ZY-S, 1.2–1.4 mm, Sigmund Lindner GmbH, Warmensteinach, Germany) in two 30-s cycles with an intermediate 30-s pause. The resulting tissue homogenate (100 μl) was mixed with 20 μl of internal standards mixture (Supplementary Table S2) and methanol (200 μl). Next, 1000 µl of methyl *tert*-butyl ether was added, followed by mixing for 10 min. Subsequently, 250 µl of water was added, and the mixture was centrifuged at 16 873*g* for 10 min. For cultivation media samples, 280 µl of media solution was mixed with 10 μl of the internal standards mixture, 1000 µl of methyl *tert*-butyl ether, and 300 µl of methanol. These samples were vortexed for 10 min, then centrifuged under the same conditions as the tissue extracts. In both cases, the organic (upper) layer was carefully collected (500 μl for explants, and 1000 µl for media), evaporated using a vacuum concentrator (Eppendorf, Hamburg, Germany), and reconstituted in 500 µl of hexane–propan-2-ol–chloroform (2:1:1, v/v) mixture before SFC–MS analysis.

### SFC–MS conditions

SFC–MS lipidomic analysis was performed according to our previously published procedure (Lísa and Jiránková, 2022) with an Acquity UPC^2^ system coupled to a Vion IMS QTOF mass spectrometer (Waters Corporation, Milford, MA, USA) using a Torus Diol column (100 × 3.0 mm, 1.7 μm, Waters) at 60°C with a flow rate of 1.7 ml/min, active back pressure regulator set to 11.03 MPa, an autosampler temperature of 8°C, an injection volume of 1 μl, and gradient of carbon dioxide (A) and methanol–propan-2-ol–water (69:30:1, v/v) with 10 mM ammonium formate (B): 0 min—1% B, 7 min—50% B, 7.2 min—1% B, 10 min—1% B. The injector needle was washed with a hexane–propan-2-ol–water (2:2:1, v/v) mixture after each injection. Positive- and negative-ion electrospray ionization (ESI) full-scan mass spectra were acquired in the sensitivity mode with the following parameters: make-up liquid of methanol at a flow rate of 0.35 ml/min, mass range m/z 50–1000, scan time 0.15 s, desolvation temperature 650°C, desolvation gas flow 1000 L/h, source offset 80 V, source temperature 120°C, cone gas flow 50 L/h, cone voltage 40 V, and capillary voltage 3 kV and 2.2 kV for positive- and negative-ion ESI, respectively. Leucine enkephalin was used as the lock mass for all experiments. Lipid species were annotated based on the tandem mass spectra of lipid class representatives, retention times of lipid class standards, retention behavior of species differing in acyl chains, and their accurate m/z (<5 ppm) in ESI mass spectra.

### RNA isolation, reverse transcription, and quantitative PCR analysis

Total RNA was isolated from 100 mg of placental explant tissue using Tri Reagent solution according to the manufacturer’s instructions. The purity and concentration of the isolated RNA were verified by measuring the absorbance ratios using a NanoDrop 1000 Spectrophotometer (Thermo Fisher Scientific). Reverse transcription was performed using the iScript Advanced cDNA Synthesis Kit and T100 Thermal Cycler (Bio-Rad, Hercules, CA, USA). qRT-PCR analysis of gene expression was performed using QuantStudio 6 (Thermo Fisher Scientific). cDNA (12.5 ng/μl) was amplified using the TaqMan™ Fast Advanced Master Mix (Thermo Fisher Scientific) in a total reaction volume of 5 μl/well with predesigned TaqMan Real-Time Expression PCR assays (Supplementary Table S3), following the thermal program specified in the manufacturer’s instructions. The relative gene expression was normalized against the geometric mean expression of the four reference genes (Supplementary Table S3).

### Quantification of cytokines in explant supernatants

Pro-inflammatory cytokine levels in the explant supernatants were measured using highly sensitive ELISA kits (Thermo Fisher Scientific). The cytokines analyzed included IL-1β (catalog no. BMS224-2), TNF-α (catalog no. KHC3011), and IL-6 (catalog no. KHC0061). All assays were performed according to the manufacturer’s protocols.

### Western blot analysis

Protein expression analysis was performed on explant tissue homogenates prepared in lysis buffer containing 50 mM Tris–HEPES (pH 7.2), 5 mM EGTA, 5 mM EDTA, 1 mM phenylmethylsulfonyl fluoride, and 250 mM sucrose. Protein concentrations were determined using the Pierce BCA Protein Assay Kit, following the manufacturer’s instructions.

Aliquots of homogenates (55 μg total protein) were mixed with LDS loading buffer under reducing conditions, heated at 70°C for 10 min, and separated by SDS–PAGE on 10–12% Bis-Tris gels using the mPAGE^®^ Lux Casting System (Merck). Electrophoresis was conducted at 120 V, and the proteins were transferred to PVDF membranes (Bio-Rad). Membranes were blocked for 1 h at room temperature in TBS-T buffer (20 mM Tris-HCl–pH 7.6, 150 mM NaCl, 0.1% Tween 20) containing 5% bovine serum albumin and then washed with TBS-T.

Primary antibody incubation (Supplementary Table S4) was carried out overnight at 4°C, followed by washing and incubation with the appropriate secondary antibodies for 1 h at room temperature. The membranes were developed using the ECL Prime Western Blotting System (Cytiva, Marlborough, MA, USA). Protein bands were visualized and quantified using the ChemiDoc MP Imaging System (Bio-Rad). To confirm equal loading, membranes were reprobed with reference protein antibodies and corresponding secondary antibodies (Supplementary Table S4).

### Data processing and statistical analysis

Peak areas of lipid species were corrected based on the isotopic pattern using Excel macro script LipidQuant 1.0 (Wolrab *et al*., 2021). Concentrations of lipids were calculated from the corrected peak areas using lipid class internal standard (Supplementary Table S2). Zero values were replaced by two-thirds of the minimum value determined in each lipid class. *P*-values were calculated using the Wilcoxon test for pairwise comparisons and the paired Friedman test for comparisons involving more than two groups. The fold change was calculated as the ratio of the mean of the treated group to the mean of the control group. The statistical output underlying the volcano plots, including fold change values and *P*-values adjusted for multiple testing using Bonferroni correction, has been deposited in an open-access repository together with the raw lipidomics dataset (see Data Availability Statement).

Heatmaps of gene expression data, presented as log_2_ fold changes, were prepared to visualize the effects of treatments on gene expression. For gene expression data, a fold change ≥2 or ≤0.5 (log_2_ fold change ≥1 or ≤−1) was considered significant. Statistical differences in protein expression and viability assays were analyzed using mixed-effects analysis with the Geisser–Greenhouse correction followed by Dunnett’s multiple comparisons test, or repeated-measures one-way ANOVA with the Geisser–Greenhouse correction, followed by Sidak’s multiple comparisons test, as appropriate depending on data structure and completeness.

All statistical analyses were performed in Prism software (GraphPad Software, Boston, MA, USA; version 9.0 or 10.0), and statistical significance was defined as *P* < 0.05. The circular heatmap was prepared using the pyCirclize package in Python software (Python Software Foundation, Wilmington, DE, USA).

## Results

### Lipidomic profiling of placental tissue, cultured explants, and explant supernatants

To characterize the intrinsic lipid composition of the placental explant model, lipidomic profiling was performed on term placental tissue homogenates, cultured explants, and explant culture supernatants using SFC–MS. A total of 204 lipid species were annotated across nine lipid classes: cholesteryl esters (CE), free fatty acids (FA), triacylglycerols (TG), diacylglycerols (DG), cholesterol (Chol), ceramides (Cer), phosphatidylcholines (PC), phosphatidylethanolamines (PE), and sphingomyelins (SM). Lipid concentrations were calculated following isotopic correction, using internal standards added during sample preparation (Supplementary Table S2).

**Figure 1.**
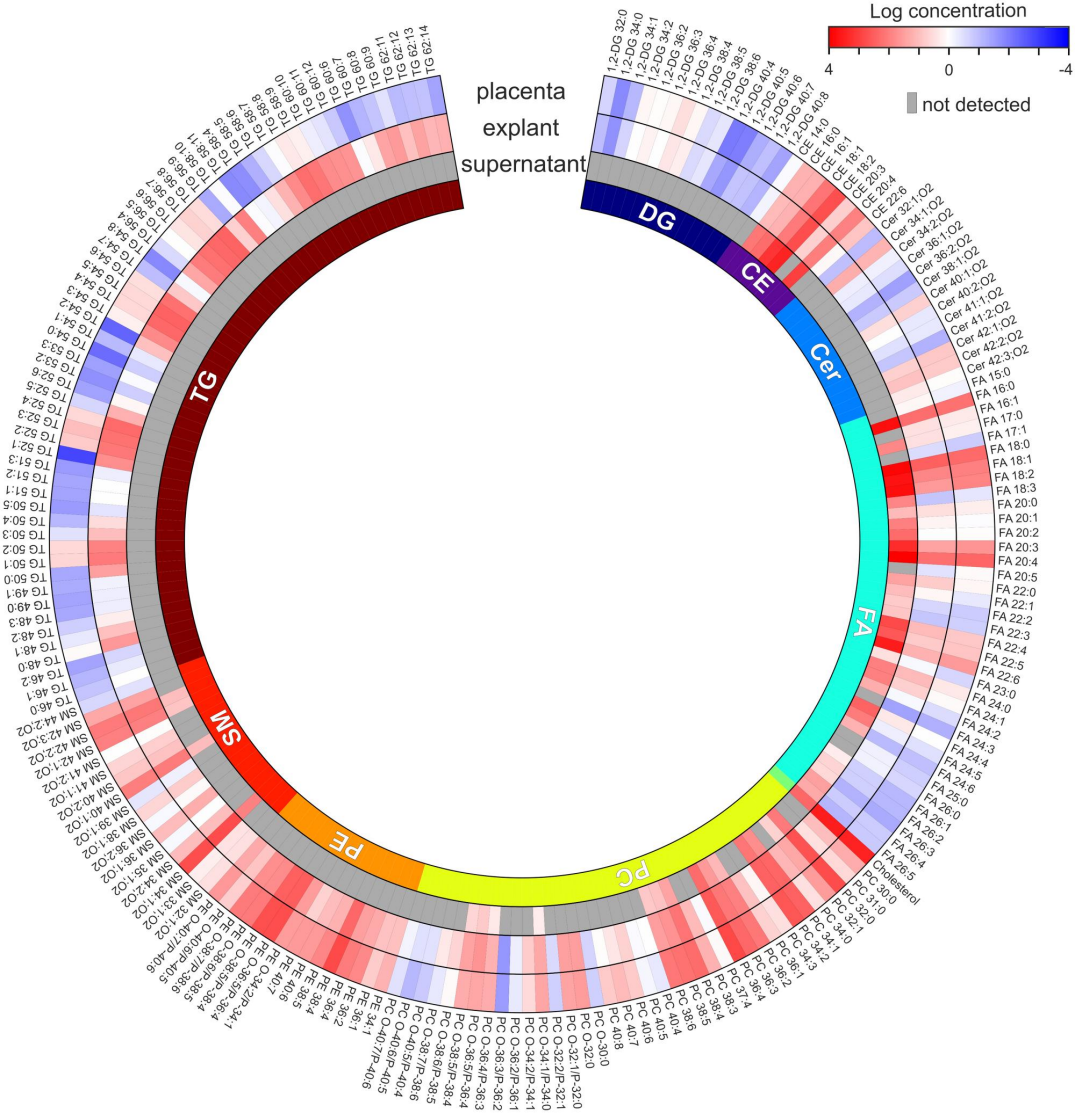
**Circular heatmap of lipids annotated in placental tissue, cultured explants, and explant supernatant samples.** The heatmap visualizes the distribution of lipid species across placental homogenates (ng/mg), cultured explants (ng/mg), and supernatant samples (ng/ml) using a logarithmic scale of mean concentrations. Grey areas indicate undetected lipid species. Healthy human placental explants were incubated for 24 h in delipidated media. Lipidomic analysis was performed using supercritical fluid chromatography coupled with mass spectrometry (SFC–MS); n = 8 biological replicates. CE, cholesteryl esters; Cer, ceramides; Chol, cholesterol; DG, diacylglycerols; FA, free fatty acids; PC, phosphatidylcholines; PE, phosphatidylethanolamines; SM, sphingomyelins; TG, triacylglycerols.

The lipid profile of placental tissue exhibited a broad distribution of species across all nine lipid classes and served as a reference for comparison (Table 1). Cultured explants retained a lipidomic profile closely resembling that of the tissue homogenates, with comparable class distributions. In terms of lipid species diversity, TG, PC, and FA were the most represented classes in explants, comprising 60, 38, and 37 detected species, respectively. In contrast, total lipid concentrations were dominated by cholesterol (3305 ng/mg) and TG (3303 ng/mg), followed by PC (Table 1). Analysis of the culture supernatants revealed lipid species released by the explants during incubation. After subtracting background levels present in the delipidated media (media blank), the supernatants were found to contain secreted FA (30 species), PC (15 species), CE (5 species), SM (4 species), and cholesterol. A circular heatmap (Fig. 1) illustrates the distribution and relative abundance of lipid species across the placental tissue, cultured explants, and explant supernatants.

**Table 1.** Total concentrations of lipid classes in placental tissue, cultured explants, and explant supernatants.

Lipid class	Placental tissue (ng/mg)	Cultured explants (ng/mg)	Explant supernatants (ng/ml)
**DG**	10	9	–
**CE**	806	824	5454
**Cer**	38	41	–
**FA**	905	893	42 320
**Chol**	2847	3305	223
**PC**	3229	2144	630
**PE**	2470	1167	–
**SM**	978	1049	119
**TG**	67	3303	–

Values are expressed as ng/mg for placental tissue and cultured explants, and ng/ml for explant supernatants. “–” indicates not detected.

CE, cholesteryl ester; Cer, ceramide; Chol, cholesterol; DG, diacylglycerol; FA, fatty acid; PC, phosphatidylcholine; PE, phosphatidylethanolamine; SM, sphingomyelin; TG, triacylglycerol.

### Lipidomic changes in placental explants following treatment with high glucose, LPS, and metformin

**Figure 2.**
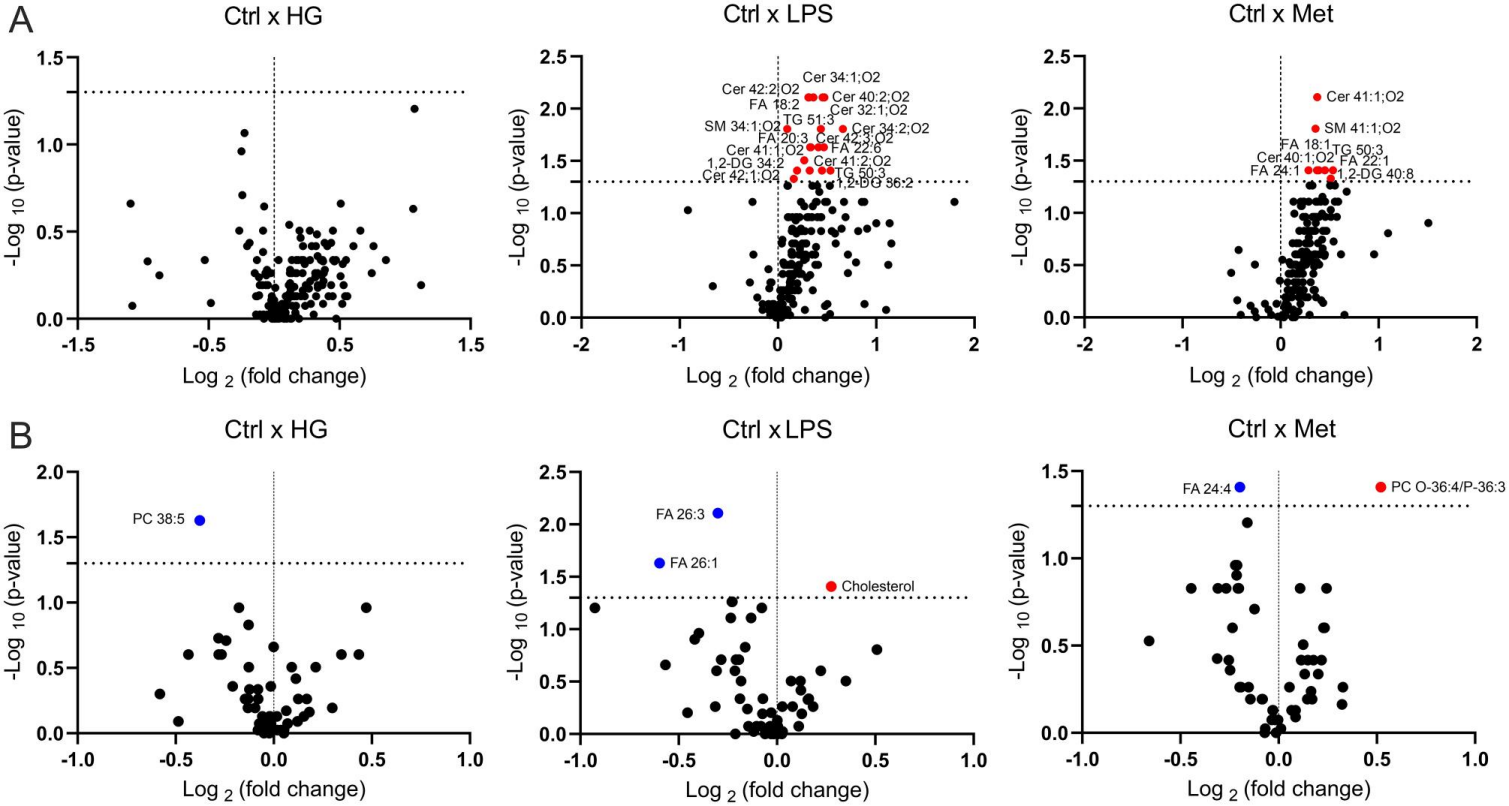
**Volcano plots illustrating the effects of treatments on lipid concentrations in (A) placental explants and (B) supernatant.** Healthy human placental explants were incubated for 24 h with high glucose (25 mM), lipopolysaccharide (10 µg/ml), or metformin (100 µM). Lipidomic analysis of explant tissue and media was performed using supercritical fluid chromatography coupled with mass spectrometry (SFC–MS). *P*-values were calculated using the Wilcoxon test for pairwise comparisons, and fold changes represent the ratio of mean lipid concentrations in the treated group relative to the control group; n = 8 biological replicates. Color coding: red, upregulated lipids; blue, downregulated lipids; black, unchanged.

To investigate how metabolic, microbial, and pharmacological stimuli influence placental lipid composition, we performed lipidomic profiling of explant tissues and corresponding culture media following treatment with high glucose, LPS, or metformin. The effects of each treatment are visualized in volcano plots, highlighting lipid species with significantly altered concentrations compared to controls (Fig. 2).

In placental explants (Fig. 2A), exposure to excess glucose (25 mM) had minimal impact on the lipidome, with no major shifts detected across lipid classes. In contrast, metformin (100 µM) selectively increased the abundance of several lipid species, including ceramides (Cer 41:1; O2, Cer 40:1; O2), free fatty acids (FA 18:1, FA 22:1, FA 24:1), triacylglycerol (TG 50:3), diacylglycerol (1,2-DG 40:8), and sphingomyelin (SM 41:1; O2). The most pronounced changes were observed following LPS treatment (10 µg/ml), which induced broad lipid remodeling consistent with an inflammatory response. Specifically, LPS significantly increased multiple ceramides (e.g. Cer 32:1; O2, Cer 34:1; O2, Cer 34:2; O2, Cer 40:2; O2, Cer 41:1; O2, Cer 41:2; O2, Cer 42:1; O2, Cer 42:2; O2, Cer 42:3; O2), as well as sphingomyelins (SM 41:1; O2), free fatty acids (FA 18:2, FA 20:3, FA 22:6), diacylglycerols (1,2-DG 34:2, 1,2-DG 36:2), and TG 50:3 and TG 51:3.

In the corresponding culture supernatants (Fig. 2B), treatment effects were more limited. High glucose led to a slight reduction in phosphatidylcholine (PC 38:5), while metformin increased the release of ether/plasmalogen-linked phosphatidylcholine (PC O-36:4/P-36:3) and decreased the release of FA 24:4. LPS significantly elevated cholesterol levels in the medium, while concurrently reducing the concentrations of certain long-chain fatty acids (FA 26:1, FA 26:3).

### Inflammatory responses to high glucose and LPS in placental explants

**Figure 3.**
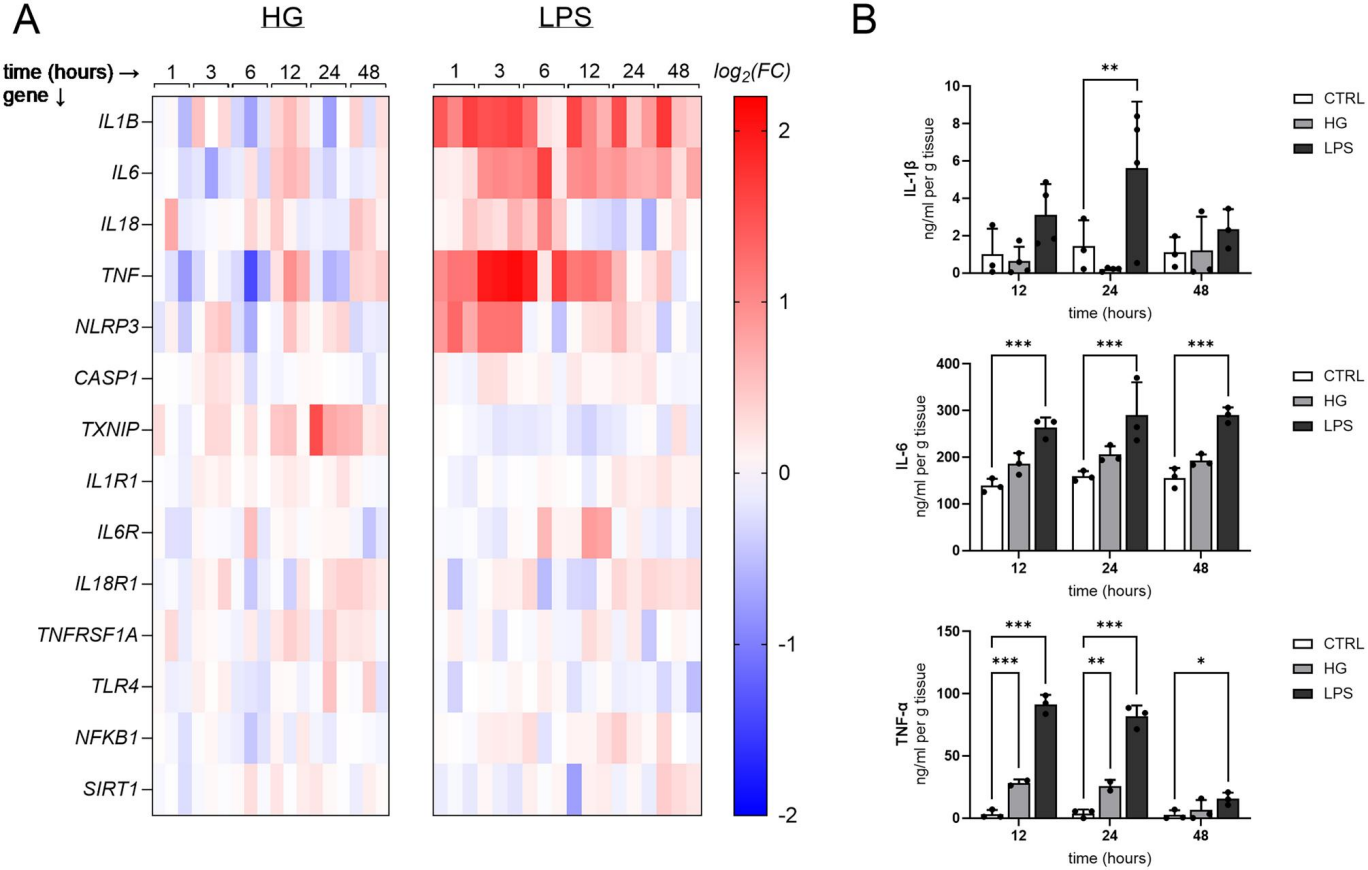
**Time-dependent effects of high glucose (HG) and lipopolysaccharide (LPS) on gene expression and cytokine release in human term placental explants.** (**A**) Heatmap illustrating the time-dependent effects of HG (35 mM) and LPS (10 µg/ml) on the expression of cytokines, inflammasome-related genes, and their receptors in placental explants. Columns represent individual biological replicates grouped by treatment and exposure time (1, 3, 6, 12, 24, and 48 h; n = 3 per condition), and rows represent individual genes. Data are presented as log_2_ fold change compared to control. (**B**) Time-dependent release of pro-inflammatory cytokines IL-1β, IL-6, and TNF-α in placental explant supernatants treated with HG (35 mM) or LPS (10 µg/ml). Cytokine concentrations in the supernatant were measured at 12-, 24-, and 48-h using ELISA. Explants cultured in standard media served as the control. Statistical differences were analyzed using mixed-effects analysis with the Geisser-Greenhouse correction, followed by Dunnett’s multiple comparisons test. Data are presented as mean + SD with individual data points shown; n = 3–4. Statistical significance is denoted as follows: *P < 0.05, **P < 0.01, ***P < 0.001.

The inflammatory effects of high glucose and LPS were assessed by measuring both cytokine production and the expression of key inflammatory and inflammasome-related mediators. In time-dependent studies, exposure to 35 mM glucose resulted in only a mild effect on gene expression, with slight upregulation of *IL1B* and *IL6* observed at 12 h (Fig. 3A). Notably, high glucose also modestly increased thioredoxin-interacting protein (*TXNIP)* expression, most evident at 24 h (Fig. 3A). In contrast, LPS (10 µg/ml) elicited a robust and rapid response. Significant upregulation was observed in cytokine-related genes (*IL1B*, *IL6*, *TNF*, and *IL18*) as well as inflammasome-associated genes (*NLRP3*). These changes were evident as early as 1 h and peaked between 1 and 12 h (Fig. 3A). Moreover, the time-dependent release of IL-1β, IL-6, and TNF-α proteins into the supernatant closely mirrored the gene expression patterns (Fig. 3B). LPS treatment (10 µg/ml) significantly increased IL-1β levels at 24 h. For IL-6, LPS induced a significant increase at all measured time points (12, 24, and 48 h). In the case of TNF-α, both LPS and high glucose (35 mM) treatments significantly elevated its levels at 12 and 24 h; however, this effect was diminished by 48 h.

In concentration-dependent experiments, glucose exposure across a range of concentrations (10–35 mM) elicited only modest and inconsistent changes in inflammatory marker expression. In contrast, LPS produced a robust response even at the lowest dose tested (0.1 µg/ml), with significant and consistent upregulation of *IL1B*, *IL6*, *TNF*, *IL18*, and *NLRP3* across all concentrations examined (0.1, 1, and 10 µg/ml) (Supplementary Fig. 2B). To determine whether the weak response to high glucose was related to osmotic stress rather than a metabolic effect, parallel experiments were performed using mannitol as an osmotic control. The inflammatory gene expression patterns observed with mannitol mirrored those seen with high glucose across comparable concentrations (Supplementary Fig. 2A).

### Differential modulation of placental inflammatory responses by metformin with and without LPS

To investigate the modulatory effects of metformin under inflammatory and non-inflammatory conditions, two experimental approaches were applied. In the first, placental explants were pretreated with increasing concentrations of metformin (0.1–1000 µM) for 24 h., followed by co-exposure to metformin and LPS (1 µg/ml) for 6 h. In the second, explants were treated with metformin alone (1 mM) for 24 or 48 h to evaluate its time-dependent effects in the absence of inflammatory stimuli.

**Figure 4.**
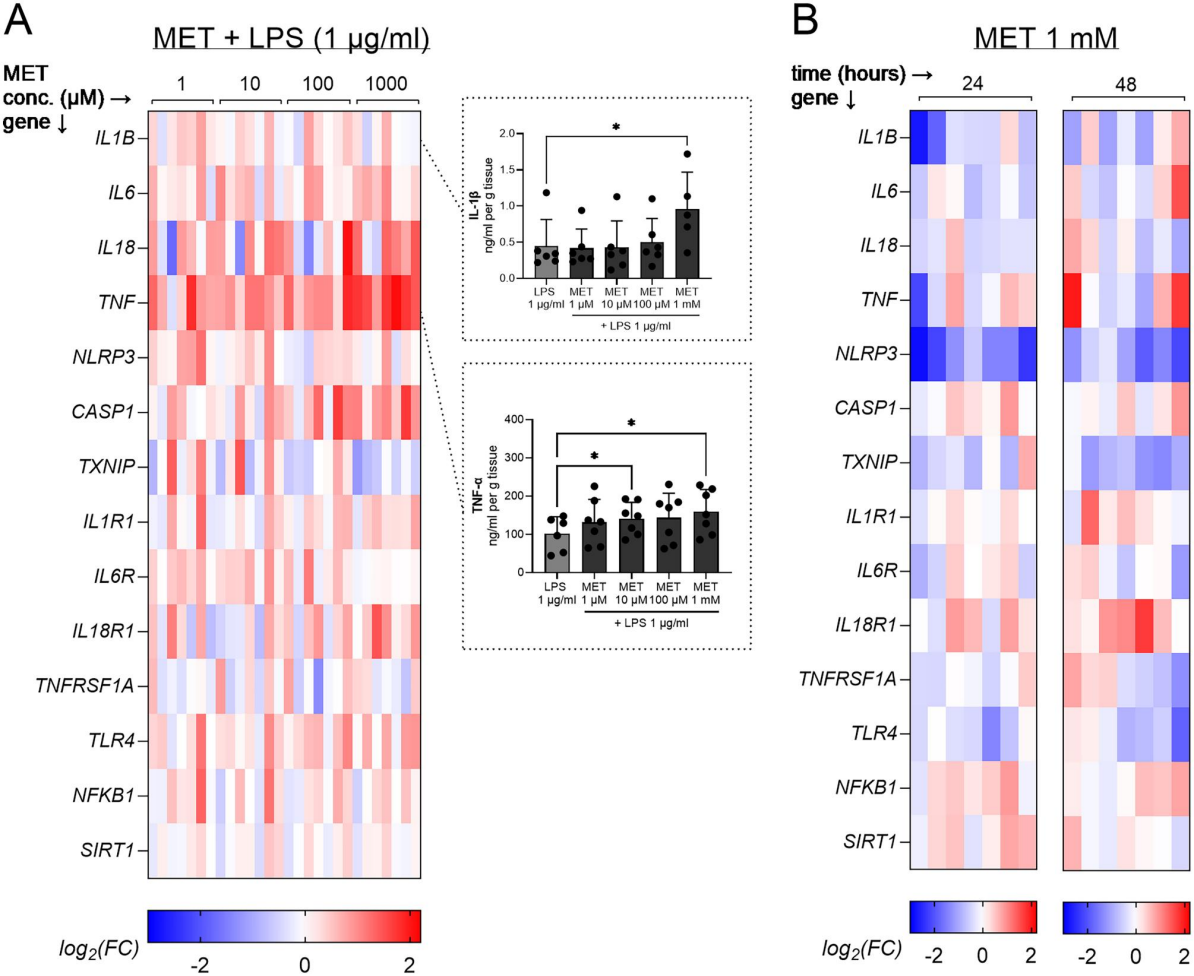
**Modulatory effects of metformin on lipopolysaccharide (LPS)-induced inflammatory responses and time-dependent effects of metformin in human term placental explants.** (**A**) Effects of metformin pretreatment on LPS-induced gene expression and cytokine release. Explants were pretreated with metformin at varying concentrations for 24 h, followed by exposure to LPS (1 µg/ml) for 6 h. Columns represent individual biological replicates grouped according to metformin pretreatment concentration (1, 10, 100, and 1000 µM; n ≥ 6 per condition), and rows represent individual genes. Data are expressed as log_2_ fold changes relative to the control group treated with LPS alone for 6 h without metformin pretreatment or as mean + SD with individual data points shown. (**B**) Time-dependent effects of metformin (1 mM) alone on gene expression. Columns represent individual biological replicates, grouped by metformin treatment duration (24 and 48 h; n ≥ 6 per condition), and rows represent individual genes. Data are expressed as log_2_ fold changes relative to a control group without metformin treatment. Statistical analysis for protein data (A) was performed using mixed-effects analysis with the Geisser-Greenhouse correction, followed by Dunnett’s multiple comparisons test to compare treated groups to LPS-treated or untreated controls. Statistical significance is denoted as follows: *P < 0.05.

When combined with LPS, metformin pretreatment resulted in a concentration-dependent increase in the expression of several inflammatory genes, with *TNF* expression significantly elevated at the highest dose (1 mM) compared to LPS alone (Fig. 4A). At the protein level, metformin also enhanced TNF-α release into the culture medium, reaching significance at both 10 µM and 1 mM. In contrast, metformin alone led to a modest downregulation of inflammatory gene expression over time, including a significant reduction in *NLRP3* mRNA at both 24 and 48 h (Fig. 4B). However, this transcriptional effect was not accompanied by changes at the protein level. Western blot analysis revealed no significant alterations in NLRP3, apoptosis-associated speck-like protein containing a CARD (ASC), or pro-CASP1 expression following metformin treatment alone or in combination with LPS (Supplementary Fig. S3). Full-length immunoblots are presented in Supplementary Fig. S4.

## Discussion

Our lipidomic profiling of term placental explants demonstrates that these cultures preserve both the lipid complexity and secretory competence of intact placental tissue, reinforcing their utility as an *ex vivo* model for studying placental lipid homeostasis. Compared to placental tissue, explants exhibited a notable enrichment of TG, which may represent transient lipid storage pools formed during the early culture period. This is in line with pulse-chase experiments by Coleman and Haynes, who described intracellular TG turnover as a central component of FA secretion (Coleman and Haynes, 1987). The observed accumulation at 24 h differs from the reduction in TG content described in primary trophoblast cells after prolonged culture (Ferchaud-Roucher *et al.*, 2017), pointing toward syncytialization-dependent shifts in lipid handling. While our explants are primarily composed of syncytiotrophoblast, viable cytotrophoblasts likely remain metabolically active (Miller *et al.*, 2005) and may contribute to lipid anabolism and membrane remodeling, as previously proposed (Kolahi *et al.*, 2017). This cellular configuration, combined with the relatively early time point, may explain the TG enrichment we observe.

In addition to intracellular accumulation, we detected the release of several lipid classes into the culture medium. Beyond FA, explants secreted PC, CE, SM, and cholesterol, suggesting a more complex pattern of lipid export than often appreciated. Given the hydrophobic nature of many lipids and the use of only 10% delipidated serum, solubility in the aqueous medium may have been limited, potentially affecting extracellular lipid recovery. Nonetheless, the pattern of lipid release did not mirror the full spectrum of intracellular lipids, indicating that export is likely selective and tightly regulated rather than merely a reflection of passive diffusion, as also discussed by Thornburg *et al.* (2019). This aligns with prior observations of restricted maternal–fetal lipid transfer (Gázquez *et al.*, 2019) and supports the concept of the placenta acting as a metabolic gatekeeper, even under *ex vivo* conditions. Among the secreted lipids, the presence and LPS-induced increase of FA 22:6 (DHA) is notable. Considering the importance of n-3 FA transfer for fetal neurodevelopment (Thornburg *et al.*, 2019), this finding suggests a potential mechanism by which inflammation may interfere with essential FA supply to the fetus. To our knowledge, this is the first study to demonstrate selective lipid secretion, including DHA, from human term placental explants under defined *ex vivo* conditions.

Exposure to high glucose did not markedly alter placental lipid composition, in contrast to findings in serum-containing systems where increased TG were observed (Hulme *et al.*, 2019). Our results more closely align with those of Watkins *et al.* (2022), who used a serum-free system and reported no TG accumulation in response to high glucose, but instead noted altered arachidonic acid flux using isotopically labeled precursors. These differences highlight the importance of media composition and lipid availability in shaping placental lipid responses to glucose. They also suggest that acute high-glucose exposure in term explants is insufficient to recapitulate the lipidomic shifts observed *in vivo* (Lu *et al.*, 2016), where chronic maternal hyperglycemia and systemic factors likely play a major role.

Several studies suggest that hyperglycemic stress can promote inflammatory signaling in placental tissue via proinflammatory cytokine production and NLRP3 inflammasome activation. Placental samples from pregnancies complicated by diabetes, including GDM and type 2 diabetes mellitus, exhibit increased IL-1β, IL-6, and inflammasome components (Corrêa-Silva *et al.*, 2018), while explant and cell-based models report glucose-induced IL-6, IL-1β, and TNF-α production, though often only at very high concentrations (Han *et al.*, 2015; Jimenez-Escutia *et al.*, 2023). Mechanistically, hyperglycemia elevates TXNIP, which, upon reactive oxygen species (ROS)-mediated thioredoxin (TRX) oxidation, dissociates to activate NLRP3 (Schroder *et al.*, 2010; Koenen *et al.*, 2011; Feng *et al.*, 2016). TXNIP overexpression has been reported in placentas from GDM pregnancies (Sarina *et al.*, 2019; Gu *et al.*, 2024), supporting a potential role for this pathway in placental responses to metabolic stress.

In our model, high glucose concentration increased *TXNIP* expression, but this was not accompanied by strong IL-1β or IL-6 responses, suggesting that a second trigger, such as ROS, may be needed for full inflammasome activation (Zhou *et al.*, 2010). Moreover, the similar effects observed with mannitol imply that osmotic stress may contribute to the inflammatory response. This could involve macrophage-mediated sensing mechanisms, as tissue-resident macrophages, present in placental explants (Pavlov *et al.*, 2020), are known to detect osmolarity changes and activate the NLRP3 inflammasome (Schwartz *et al.*, 2009; Ip and Medzhitov, 2015). Overall, our findings support the concept that hyperglycemic stress may contribute to low-grade inflammatory priming in the placenta (Xuan Nguyen *et al.*, 2023), but indicate that acute glucose exposure alone is insufficient to fully activate inflammasome signaling. This highlights the requirement for longer exposure and/or additional metabolic or oxidative triggers that are likely present *in vivo*, which represents a limitation of acute stimulation models in term explants.

Consistent with infection-driven placental inflammation, LPS induced robust cytokine production and markedly altered the placental lipidome. Among the most consistent changes was the upregulation of multiple ceramide species, which mirrors findings in stressed trophoblasts (Easton *et al.*, 2023), suggesting a shared lipidomic signature of placental stress. To our knowledge, this provides the first evidence that LPS induces ceramide remodeling in human placental explants. Ceramides are bioactive sphingolipids that regulate apoptosis, autophagy, and inflammatory signaling (Pal *et al.*, 2022; Pilátová *et al.*, 2023; Augusto *et al.*, 2024). Dysregulated ceramide metabolism has been implicated in placental dysfunction, including trophoblast autophagy in preeclampsia and altered lipid homeostasis in GDM (Melland-Smith *et al.*, 2015; Ausman *et al.*, 2018; Pinto *et al.*, 2023). Our results extend these associations by showing that an acute inflammatory stimulation is sufficient to drive ceramide accumulation in the placenta. Mechanistically, ceramide accumulation is consistent with LPS–TLR4 signaling, which promotes *de novo* ceramide synthesis and can amplify downstream immune responses (MacKichan and DeFranco, 1999; Olona *et al.*, 2021). Given that ceramides have been proposed to facilitate NLRP3 inflammasome activation via ROS/TXNIP mechanisms (Scheiblich *et al.*, 2017; Jiang *et al.*, 2021; Liu *et al.*, 2022), the strong induction of both ceramides and inflammasome-related genes in our explants supports the idea that ceramide remodeling acts as a lipid signal linking innate immune activation to inflammasome assembly in the placenta.

In parallel with these lipidomic changes, LPS triggered a rapid and sustained inflammatory response at both transcriptional and protein levels. Strong upregulation of *IL1B*, *IL6*, *TNF*, and *NLRP3* transcripts was detected within hours, and this was accompanied by the release of IL-1β, IL-6, and TNF-α proteins into the culture medium. These findings align with previous reports of TLR4-driven activation of innate immune pathways in the placenta (Abad *et al.*, 2024) and support the high sensitivity of placental tissue to microbial signals, even at relatively low LPS concentrations. The close temporal overlap between cytokine release and ceramide accumulation suggests coordinated regulation of lipid and immune pathways, consistent with models in which sphingolipids act as secondary messengers amplifying inflammatory signaling (MacKichan and DeFranco, 1999; Scheiblich *et al.*, 2017; Jiang *et al.*, 2021; Liu *et al.*, 2022). Taken together, these results demonstrate that human placental explants mount a tightly coupled lipidomic and cytokine response to LPS, highlighting their value as an *ex vivo* model for investigating the crosstalk between placental metabolism and innate immunity.

While insulin remains the first-line pharmacotherapy for GDM in many international guidelines (ADA, 2025; McEvoy *et al.*, 2025), metformin is also commonly used during pregnancy as an alternative or adjunct treatment in several countries and healthcare systems, and is endorsed by some professional bodies (ADA, 2025; SMFM, 2018; Tížková, 2025). Importantly, because metformin crosses the placenta and exerts pleiotropic metabolic and immunomodulatory effects (Hur and Lee, 2015; Owen *et al.*, 2021; Xian *et al.*, 2021; Ryssdal *et al.*, 2023; Hu *et al.*, 2024; Huang *et al.*, 2024), it represents a clinically relevant pharmacological stimulus for evaluating placental explant responsiveness. In our explant model, metformin displayed a dual effect, with modest anti-inflammatory tendencies under basal conditions but paradoxical pro-inflammatory synergy when combined with LPS. When applied alone, metformin modestly downregulated transcripts associated with the NLRP3 inflammasome pathway, including *TLR4*, *IL1B*, *NLRP3*, and *TXNIP*, without major effects at the protein level. These findings are consistent with reports that metformin can dampen inflammatory signaling in different tissues by suppressing NLRP3 activation and pro-inflammatory cytokine production (Hu *et al.*, 2019; Tsuji *et al.*, 2020; Xian *et al.*, 2021; Huang *et al.*, 2024). In the placental context, this modest transcriptional effect may reflect a weak basal anti-inflammatory action, insufficient to translate into clear protein-level changes over the timeframe studied.

When explants were pretreated with metformin prior to LPS exposure, the cytokine profile shifted toward a pro-inflammatory pattern. Metformin potentiated the LPS-induced expression and release of TNF-α and IL-1β, with a similar upward trend for IL-6. This paradoxical effect aligns with reports in other systems where metformin enhanced inflammatory signaling under certain conditions, such as in women with polycystic ovarian syndrome (Ryssdal *et al.*, 2023) or during acute tissue injury (Yoon *et al.*, 2023). One possible explanation is that metformin acted as a secondary signal in the two-step model of NLRP3 activation (Broz and Dixit, 2016), enhancing cytokine release in LPS-primed explants. However, our western blot analyses did not show clear changes in inflammasome protein levels, and we did not assess cleaved caspase-1 or IL-1β, which are definitive markers of inflammasome activation. Further studies are therefore needed to establish whether metformin directly contributes to inflammasome assembly in the placenta.

The variability of metformin’s effects has also been noted in other placental models. In Sw.71 trophoblasts, metformin reduced some cytokines but not IL-1β (Han *et al.*, 2015), whereas in HTR-8/SVneo cells, it strongly suppressed NLRP3-related proteins and pyroptosis (Zhang *et al.*, 2021). Notably, Jimenez-Escutia *et al.* (2023) reported in placental explants that metformin reversed high glucose-induced cytokine upregulation but was ineffective under combined high-glucose and bacterial infection conditions. These differences likely reflect variations in treatment sequence, cellular complexity, and inflammatory context. By preserving trophoblasts, immune cells, and stromal components, explants capture a broader range of cellular interactions, which may explain the context-dependent effects we observed.

In addition to its effects on cytokine responses, metformin also altered the placental lipidome. We observed an increase in several lipid species, including Cer, SM, FA, and TG. Similar increases have been reported in primary trophoblasts after metformin exposure (Tarry-Adkins *et al.*, 2023). Conversely, we noted reduced release of certain FAs into the medium, consistent with impaired β-oxidation and lipid accumulation reported under inflammatory or metabolic stress (Visiedo *et al.*, 2023). Such changes may have implications for maternal–fetal lipid transfer, particularly in pregnancies complicated by metabolic or inflammatory diseases. Taken together, these findings highlight the complexity of metformin’s actions in the placenta and suggest that, beyond its systemic metabolic effects, it may directly influence placental immune−lipid crosstalk.

Our study has several limitations that should be considered when interpreting the findings. First, we focused exclusively on explants prepared from term placentas, which limits insight into earlier developmental stages. Recent work by Bidne et al. demonstrated gestational-stage-dependent lipid profiles, with notably higher TG and PC levels in early pregnancy (Bidne *et al.*, 2022), highlighting the importance of including first-trimester tissue in future studies to capture the developmental trajectory of placental lipid regulation. Second, our experiments involved short-term exposures under controlled *ex vivo* conditions, which do not fully replicate the chronic metabolic, hormonal, and immune milieu present *in vivo*. Third, although we observed that metformin modulated LPS-induced cytokine responses, we did not perform lipidomic profiling after combined metformin pretreatment and LPS stimulation. In addition, we did not include a graded glucose design with metformin-only and glucose ± metformin conditions. These experiments would help clarify whether metformin modifies inflammatory and lipidomic adaptations relevant to metabolic and infection-driven inflammatory stressors in pregnancy and should be addressed in future work. Finally, while we assessed inflammasome-related transcripts and proteins, we did not measure cleaved caspase-1 or IL-1β, which would have provided definitive evidence of inflammasome activation.

Taken together, human placental explants preserved key metabolic and innate immune functions *ex vivo*. Acute high glucose increased *TXNIP* but did not reproduce the broader inflammatory phenotype observed in chronic hyperglycemia *in vivo*, consistent with a requirement for chronic exposure and systemic maternal factors. LPS induced coordinated lipidomic and cytokine responses, with ceramide accumulation suggesting a possible link between TLR4 activation and inflammasome-related signaling. Metformin had modest basal effects but potentiated LPS-driven cytokine release, highlighting context-dependent immunomodulatory actions. Because lipid remodeling and inflammatory signaling are central to placental function (Thornburg *et al.*, 2019; Megli *et al.*, 2021), their disruption may contribute to adverse fetal programming and offspring outcomes. Although derived from an *ex vivo* model, our observation that metformin potentiates LPS-driven cytokine release suggests that pharmacological exposure may modify placental inflammatory responsiveness under infectious challenge. This interaction warrants validation in more integrative models and may have clinical relevance for understanding inflammatory responses in metformin-treated pregnancies when maternal infection or inflammation is present.

## Supplementary Material

gaag016_Supplementary_Data

## Data Availability

The data supporting the findings of this study are available within the article and its Supplementary Information. The raw lipidomics dataset and the statistical output used for volcano plots and multiple-testing correction have also been deposited in Zenodo (10.5281/zenodo.18214772).

## Author Biography

   **Dr. ****Rona Karahoda**: PharmDr Rona Karahoda, PhD, is an Assistant Professor at the Faculty of Pharmacy in Hradec Králové, Charles University (Czech Republic), and a member of the Placenta in Health and Disease research team. She completed both her pharmacy degree (PharmDr) and PhD in Pharmacology and Toxicology at Charles University. Her doctoral research focused on placental tryptophan metabolism and serotonin transport at the maternal–fetal interface; during this period, she undertook a research visit at the University of Bern (Switzerland). Her work integrates placental metabolism, transporter biology, and immune-inflammatory signaling in pregnancy complications, including preterm birth and gestational diabetes mellitus, and examines how prenatal exposures—such as medications and metabolic stressors—reshape placental function and fetal development. Dr Karahoda has secured competitive research funding, received scientific awards, and authored peer-reviewed publications. She remains active in the international placental research community through collaborations and conference participation.
