# Supplementary material for: Metabolic, microbial, and pharmacological stimuli elicit distinct lipidomic and cytokine responses in the human placenta
Source: Mol Hum Reprod. 2026 Mar 7;32(1):gaag016. doi: 10.1093/molehr/gaag016 (PMC13016871; doi:10.1093/molehr/gaag016)
Supplement: gaag016_Supplementary_Data [file gaag016_supplementary_data.pdf]

METABOLIC, MICROBIAL, AND PHARMACOLOGICAL STIMULI ELICIT DISTINCT LIPIDOMIC AND CYTOKINE RESPONSES IN HUMAN PLACENTA

Fiona Kumnova, Oleksandr Kozlov, Eva Cifkova, Eva Trckova, Alba Gonzalez, Michaela Medkova, Nida Cavdarbasha, Cilia Abad, Miroslav Lisa, Lukas Cerveny, Frantisek Staud, Rona Karahoda

CONTENTS PAGE

Supplementary Figure S1. Viability and integrity of human term placental explants treated with glucose, mannitol, lipopolysaccharide (LPS), and metformin.

Supplementary Figure S2. Concentration-dependent gene expression in human term placental explants treated with glucose, mannitol, and lipopolysaccharide (LPS).

Supplementary Figure S3. Protein expression of NLRP3 inflammasome components in placental explants treated with metformin, lipopolysaccharide (LPS), or both.

Supplementary Figure S4. Uncropped Western Blot images corresponding to Supplementary Figure S3.

Supplementary Table S1. Demographic characteristics of the study participants.

Supplementary Table S2. Composition of the internal standard mixture used for SFC-MS lipidomic analysis.

Supplementary Table S3. List of predesigned TaqMan Real-Time Expression PCR assays.

Supplementary Table S4. List of antibodies used in Western blot analysis.

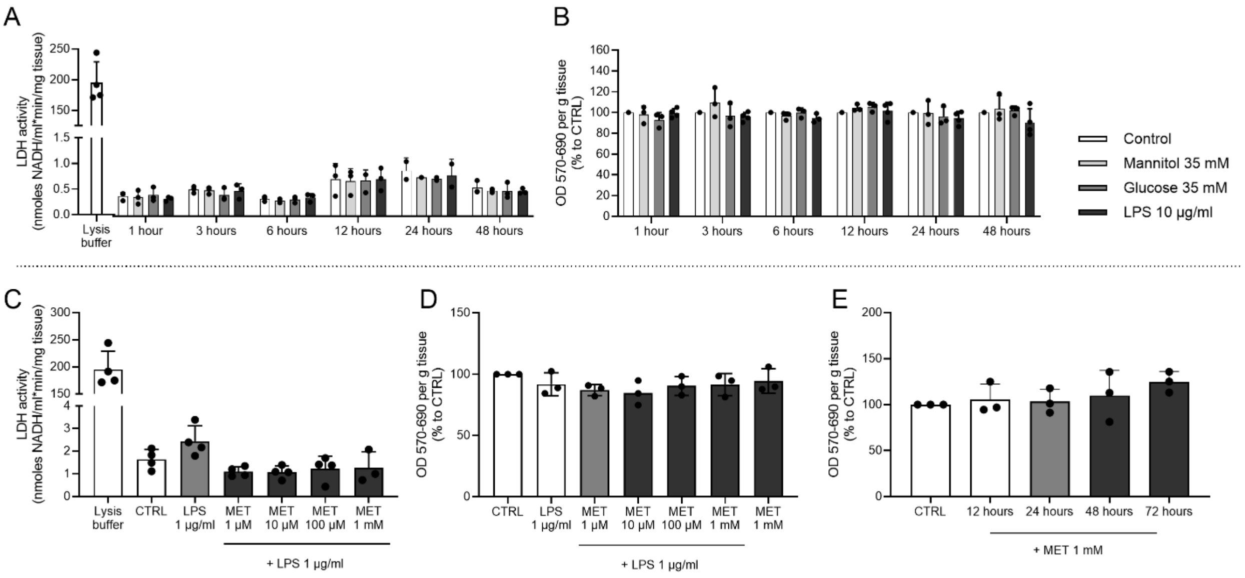

Supplementary Figure S1. Viability and integrity of human term placental explants treated with glucose, mannitol, lipopolysaccharide (LPS), and metformin. (A) Lactate dehydrogenase (LDH) activity measured in the supernatant and (B) thiazolyl blue tetrazolium bromide (MTT) assay performed on placental explants exposed to high glucose (HG; 35 mM), mannitol (MAN; 35 mM), and lipopolysaccharide (LPS; 10 µg/ml) for up to 48 hours. (C) LDH activity and (D) MTT assay results in the supernatant of explants pretreated with metformin for 24 h (MET; 1 µM–1 mM), followed by stimulation with LPS (1 µg/mL) for 6 h. (E) MTT assay results in explants exposed to 1 mM metformin for 12–72 hours. For LDH activity assays (A, C), explants treated with lysis buffer at 37°C for 15 minutes served as a positive control. Data are presented as mean ± SD with individual data points shown; n = 3 biological replicates.

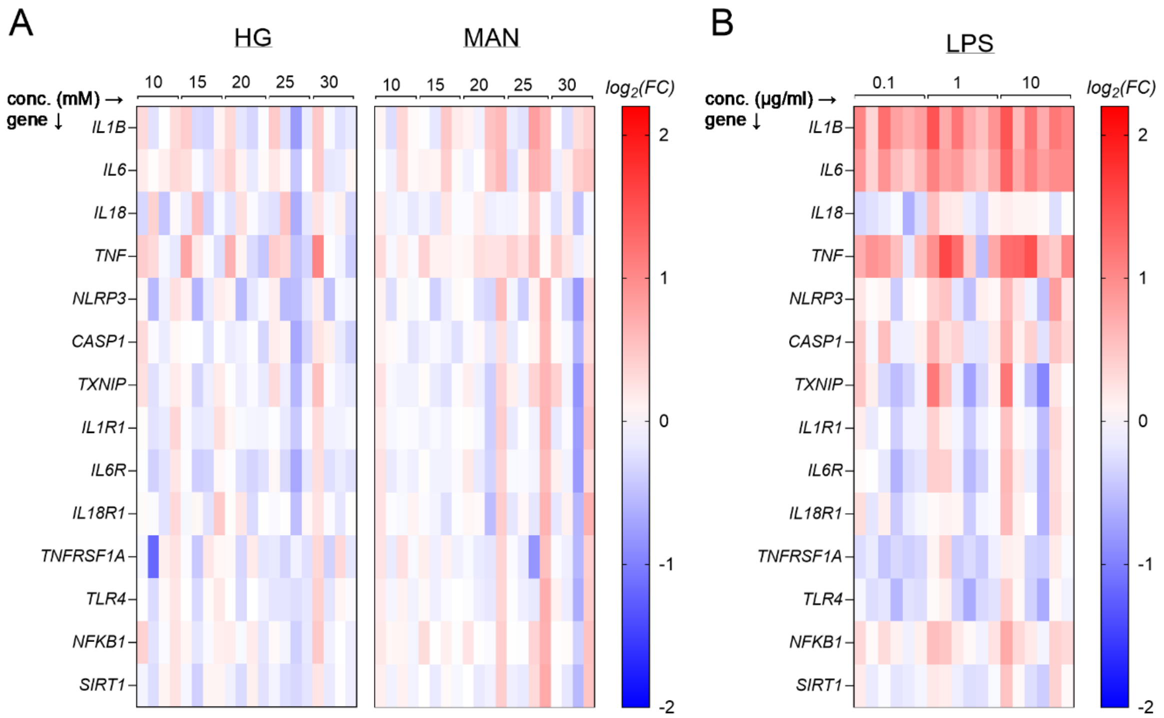

Supplementary Figure S2. Concentration-dependent gene expression in human term placental explants treated with glucose, mannitol, and lipopolysaccharide (LPS). (A) Gene expression changes in explants treated with varying concentrations of glucose (HG; 10–30 mM) and mannitol (MAN; 10–30 mM, osmotic control). (B) Gene expression changes in explants treated with varying concentrations of lipopolysaccharide (LPS; 0.1, 1, and 10 µg/ml). Samples were collected after 12 hours of treatment. Data are presented as log2 fold change compared to control; n ≥ 3 biological replicates.

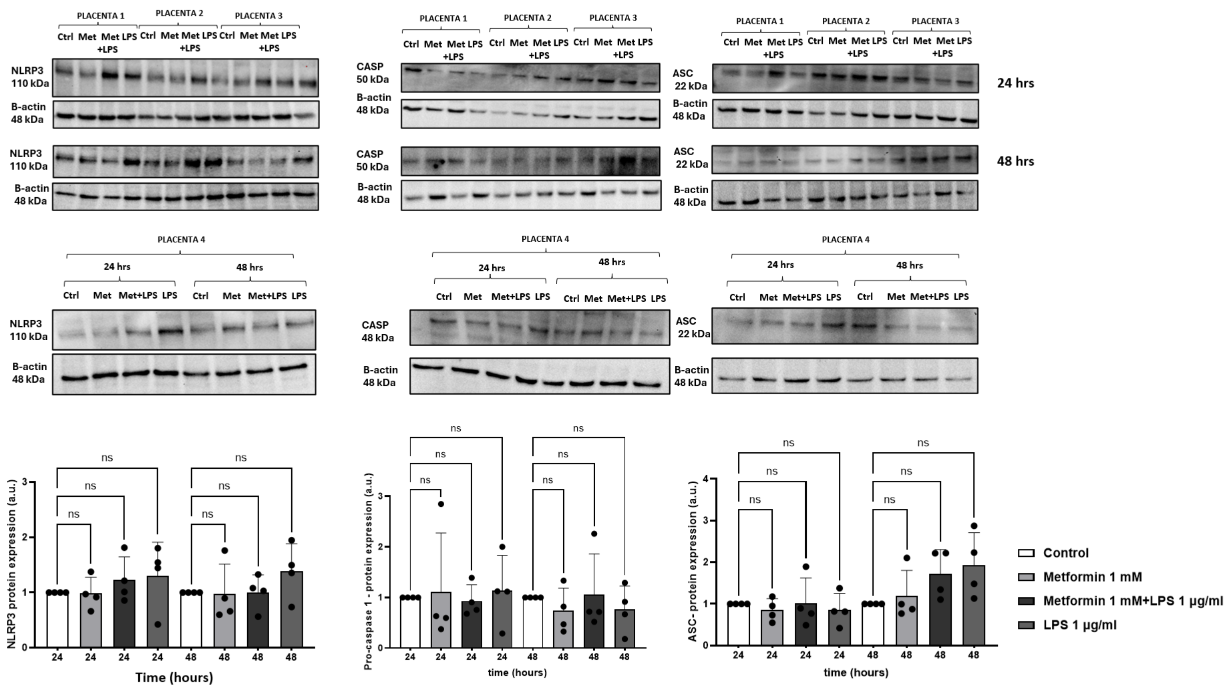

Supplementary Figure S3. Protein expression of NLRP3 inflammasome components in placental explants treated with metformin, lipopolysaccharide (LPS), or both. Explants were treated with metformin (1 mM) for 24 or 48 hours, with or without subsequent LPS exposure (1 μg/mL, 6 h). Controls included explants treated with LPS only or untreated. Western blot analysis was performed for NLRP3, ASC, and pro-caspase-1, with β-actin as the loading control. Quantification of band intensities is shown below each blot. Data are presented as mean ± SD with individual data points shown; n = 4. Statistical analysis was performed using repeated-measures ANOVA with Geisser-Greenhouse correction, followed by Sidak's multiple comparisons test; ns = not significant.

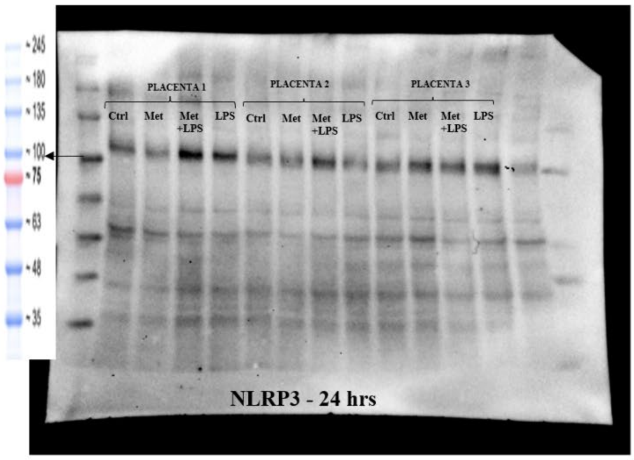
245
180
135
100
75
63
48
35
PLACENTA 1
PLACENTA 2
PLACENTA 3
Ctrl
Met
Met
+LPS
LPS
NLRP3 - 24 hrs

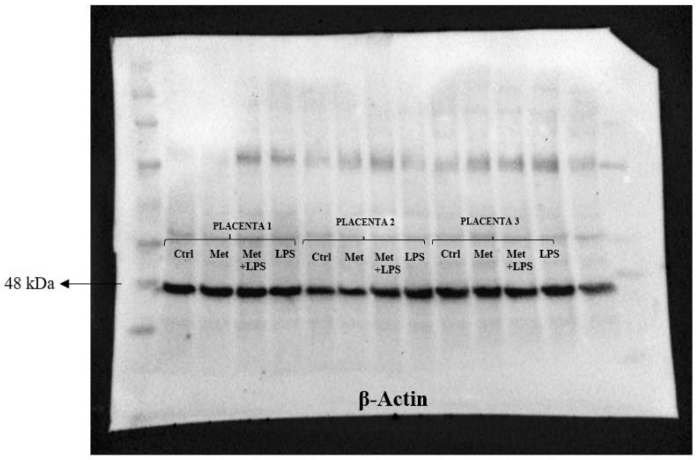
PLACENTA 1
PLACENTA 2
PLACENTA 3
Ctrl
Met
Met
+LPS
LPS
48 kDa
β-Actin

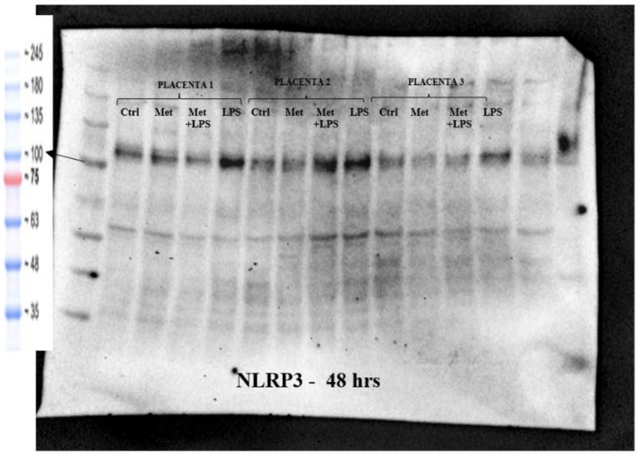
245
180
135
100
75
63
48
35
PLACENTA 1
PLACENTA 2
PLACENTA 3
Ctrl
Met
Met
+LPS
LPS
NLRP3 - 48 hrs

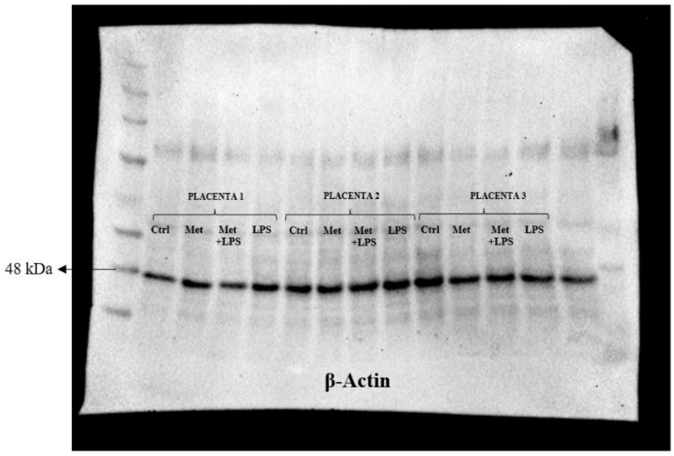
PLACENTA 1
PLACENTA 2
PLACENTA 3
Ctrl
Met
Met
+LPS
LPS
48 kDa
β-Actin

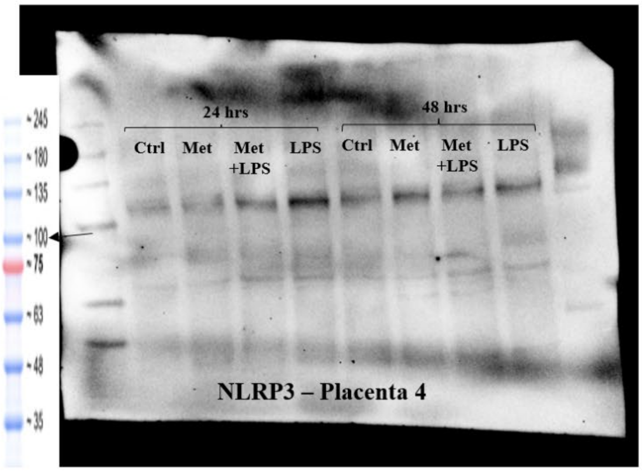
245
180
135
100
75
63
48
35
24 hrs
48 hrs
Ctrl
Met
Met
+LPS
LPS
NLRP3 – Placenta 4

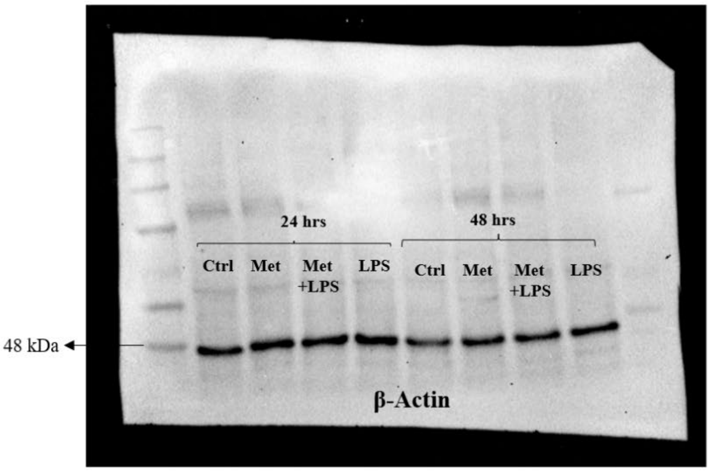
24 hrs
48 hrs
Ctrl
Met
Met
+LPS
LPS
48 kDa
β-Actin

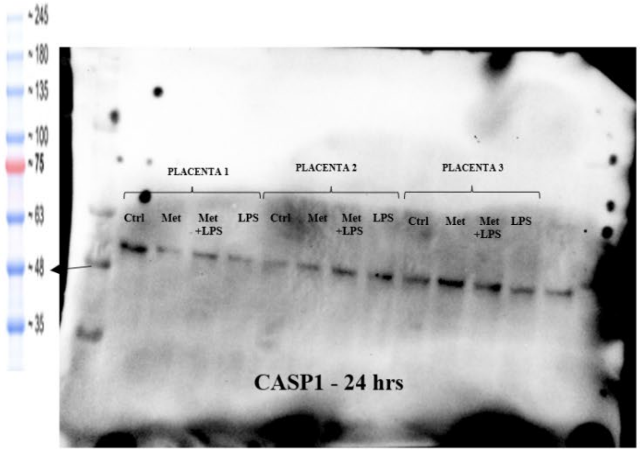
245
180
135
100
75
63
48
35
PLACENTA 1
PLACENTA 2
PLACENTA 3
Ctrl
Met
Met +LPS
LPS
Ctrl
Met
Met +LPS
LPS
Ctrl
Met
Met +LPS
LPS
CASP1 - 24 hrs

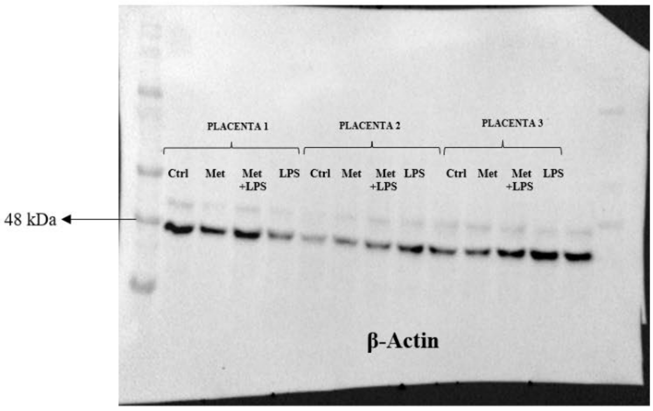
48 kDa
PLACENTA 1
PLACENTA 2
PLACENTA 3
Ctrl
Met
Met +LPS
LPS
Ctrl
Met
Met +LPS
LPS
Ctrl
Met
Met +LPS
LPS
β-Actin

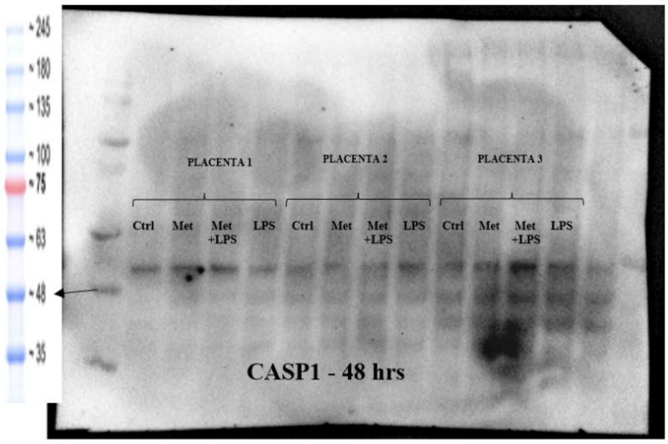
245
180
135
100
75
63
48
35
PLACENTA 1
PLACENTA 2
PLACENTA 3
Ctrl
Met
Met +LPS
LPS
Ctrl
Met
Met +LPS
LPS
Ctrl
Met
Met +LPS
LPS
CASP1 - 48 hrs

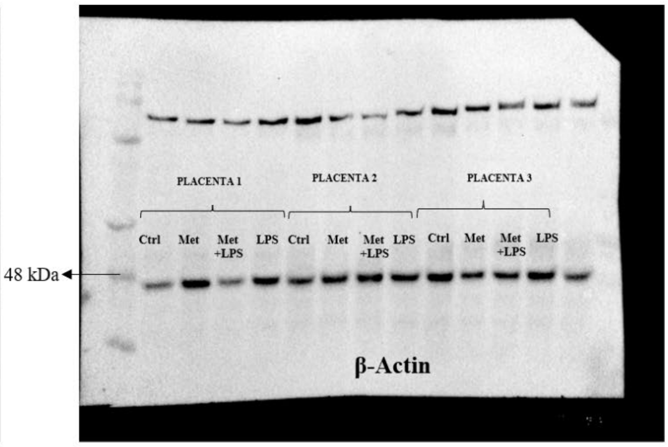
48 kDa
PLACENTA 1
PLACENTA 2
PLACENTA 3
Ctrl
Met
Met +LPS
LPS
Ctrl
Met
Met +LPS
LPS
Ctrl
Met
Met +LPS
LPS
β-Actin

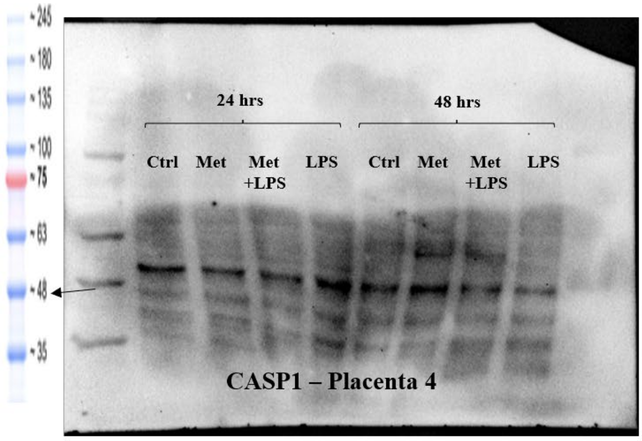
245
180
135
100
75
63
48
35
24 hrs
48 hrs
Ctrl
Met
Met +LPS
LPS
Ctrl
Met
Met +LPS
LPS
CASP1 – Placenta 4

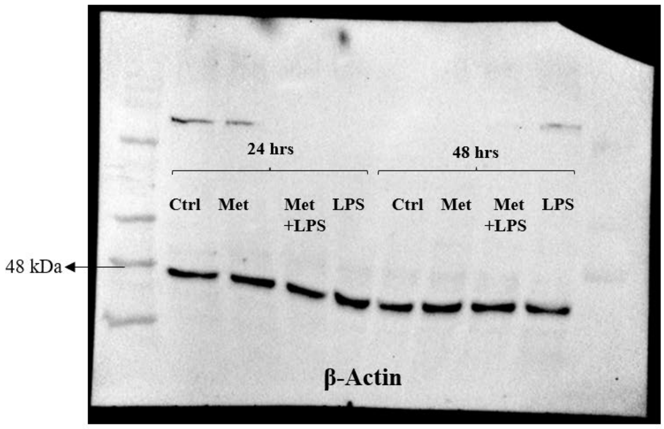
48 kDa
24 hrs
48 hrs
Ctrl
Met
Met +LPS
LPS
Ctrl
Met
Met +LPS
LPS
β-Actin

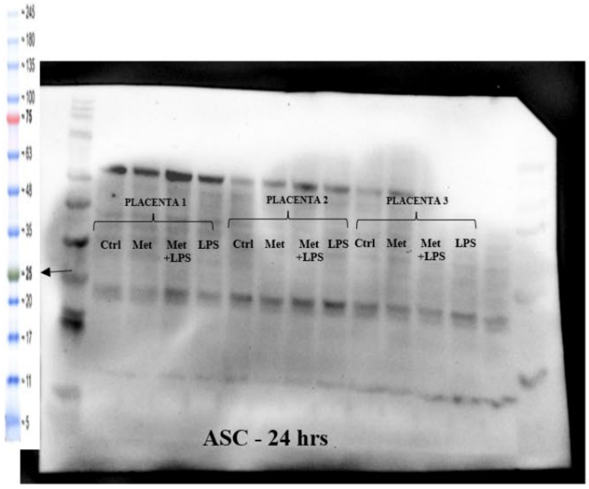

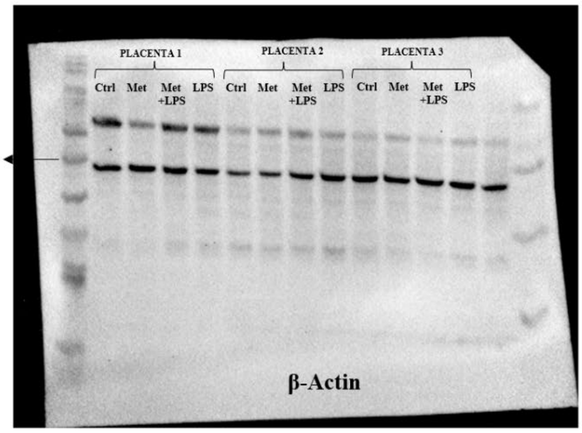

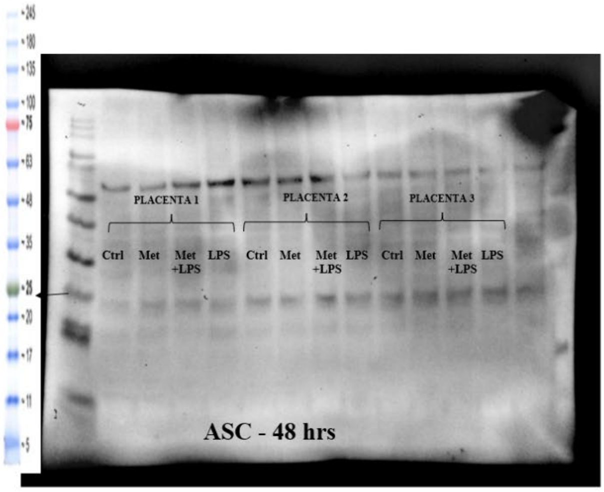

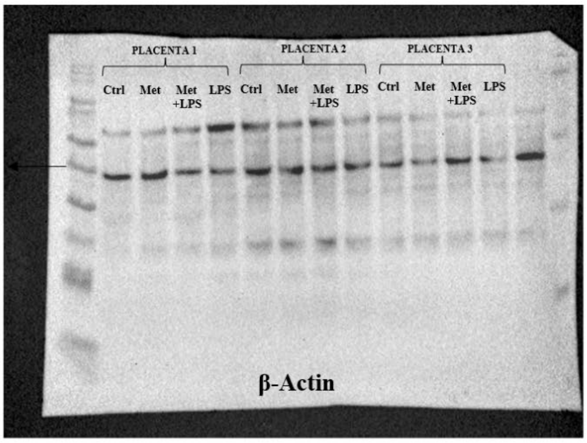

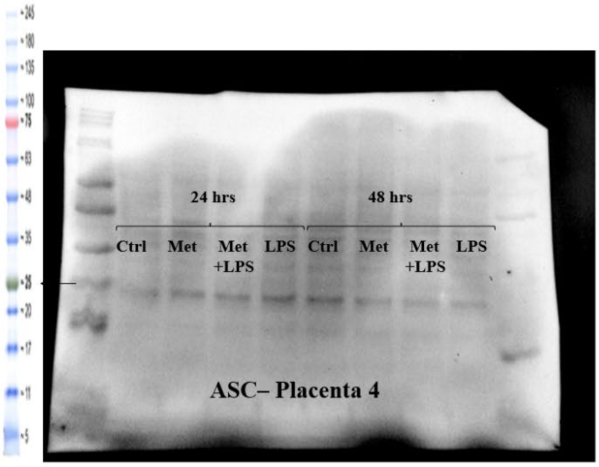

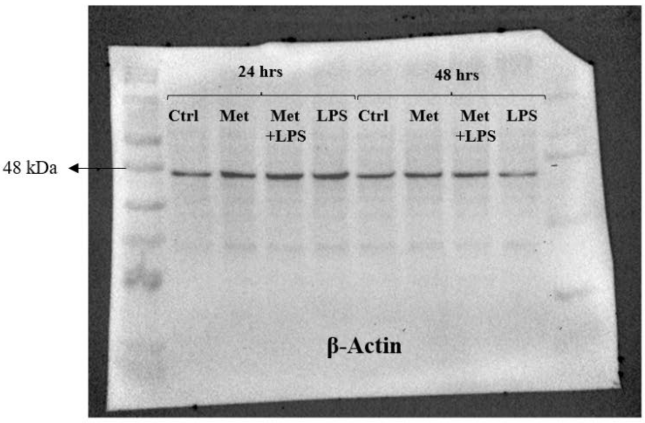

Supplementary Figure S4. Uncropped Western Blot images corresponding to Supplementary Figure S3. Explants were treated with metformin (1 mM) for 24 or 48 h, with or without subsequent lipopolysaccharide (LPS; 1 μg/mL, 6 h) exposure. Controls included explants treated with LPS only or untreated. Western blot analysis was performed for NLRP3, apoptosis-associated speck-like protein

containing a CARD (ASC), and pro-caspase-1, with β-actin used as the loading control. Abbreviations: Ctrl, control; Met, metformin (1 mM); Met + LPS, metformin (1 mM) combined with lipopolysaccharide (1 μg/mL); LPS, lipopolysaccharide (1 μg/mL).

Supplementary Table S1. Demographic characteristics of the study participants.

**Parameter**	**Term placentas (n = 28)**
Maternal age (years)	33.07 ± 5.7
Gestational age (weeks)	39.33 ± 0.94
Maternal BMI before pregnancy ($kg\ m^{-2}$)	28.51 ± 5.9
Maternal BMI after pregnancy ($kg\ m^{-2}$)	28.41 ± 5.56
Birth weight (kg)	3.52 ± 0.4
Birth height (cm)	50.18 ± 2.02
Fetal sex (F:M)	12: 16

Data are presented as mean ± SD. F, female; M, male.

Supplementary Table S2. Composition of the internal standard mixture used for SFC-MS lipidomic analysis.

**Internal standard**	**Concentration (ng/µL)**
FA 14:0	105.26
DG 12:1/0:0/12:1	1.05
TG 19:1/19:1/19:1	4.21
CE 16:0[D7]	10.53
Cholesterol[D7]	421.05
Cer d18:1/12:1	1.05
PC 14:0/14:0	42.11
PE 14:0/14:0	42.11
SM d18:1/12:0	10.53

FA, fatty acid; DG, diacylglycerol; TG, triacylglycerol; CE, cholesteryl ester; Cer, ceramide; PC, phosphatidylcholine; PE, phosphatidylethanolamine; SM, sphingomyelin; Cholesterol [D7], deuterated cholesterol internal standard; [D7], deuterium-labelled internal standard.

Supplementary Table S3. List of predesigned TaqMan Real-Time Expression PCR assays.

Gene name	Gene symbol	Assay ID
Interleukin 1β	*IL1B*	Hs 001555410_m1
Interleukin 6	*IL6*	Hs 00174131_m1
Interleukin 18	*IL18*	Hs 01038788_m1
Tumor necrosis factor α	*TNFa*	Hs 00174128_m1
NOD-, LRR-, and pyrin domain-containing protein 3	*NLRP3*	Hs 00918082_m1
Caspase-1	*CASP1*	Hs 00354836_m1
Thioredoxin-interacting protein	*TXNIP*	Hs 01006897_g1
Interleukin-1 receptor type 1	*IL1R1*	Hs 00991010_m1
Interleukin-6 receptor	*IL6R*	Hs 01075664_m1
Interleukin-18 receptor 1	*IL18R1*	Hs 00977691_m1
Tumor necrosis factor receptor superfamily member 1A	*TNFRSF1A*	Hs 01042313_m1
Toll-like receptor 4	*TLR4*	Hs 00152939_m1
Nuclear factor kappa B subunit 1	*NFKB1*	Hs 00765730_m1
Sirtuin 1	*SIRT1*	Hs 01009006_m1
Eukaryotic translation initiation factor 4A	*EIF4A*	Hs 00786996_g1
TATA-box binding protein	*TBP*	Hs 00427620_m1
DNA topoisomerase I	*TOP1*	Hs 00243257_m1
Ubiquitin C	*UBC*	Hs 05002522_g1

Supplementary Table S4. List of antibodies used in Western blot analysis.

Protein	Supplier	Catalogue no.	Dilution
ASC/TMS1	Cell Signaling Technology, Danvers, MA, USA	E1E3I	1:500
CASP1	Cell Signaling Technology, Danvers, MA, USA	E9R2D	1:500
NLPR3	Abcam, Cambridge, UK	ab263899	1:250
anti-rabbit horseradish peroxidase linked secondary antibody	Agilent Technologies, Santa Clara, CA, USA	P0217	1:2500<br>1:5000
β-actin (reference)	Abcam, Cambridge, UK	ab8226	1:10000
anti-mouse HRP-conjugated secondary antibody	Agilent Technologies, Santa Clara, CA, USA	P0260	1:20000 and 1:2500
